# Predicting Video Game Addiction Through the Dimensions of Consumer Video Game Engagement: Quantitative and Cross-sectional Study

**DOI:** 10.2196/30310

**Published:** 2021-11-26

**Authors:** Amir Zaib Abbasi, Umair Rehman, Zahra Afaq, Mir Abdur Rafeh, Helmut Hlavacs, Mohammed A Mamun, Muhammad Umair Shah

**Affiliations:** 1 Department of Management Sciences Shaheed Zulfikar Ali Bhutto Institute of Science and Technology Islamabad Pakistan; 2 Interdisciplinary Research Centers for Finance and Digital Economy, KFUPM Business School King Fahd University of Petroleum & Minerals Dhahran Saudi Arabia; 3 User Experience Design Department Wilfrid Laurier University Brantford, ON Canada; 4 Namal Institute Mianwali Mianwali Pakistan; 5 Entertainment Group University of Vienna Vienna Austria; 6 Center for Health Innovation, Networking, Training, Action and Research-Bangladesh Dhaka Bangladesh; 7 Department of Public Health and Informatics, Jahangirnagar University Savar Bangladesh; 8 Department of Management Sciences University of Waterloo Waterloo, ON Canada

**Keywords:** consumer video game engagement, dedication, absorption, social connection, interaction, conscious attention, enthusiasm, video game addiction, uses and gratifications theory, cultivation theory

## Abstract

**Background:**

Video games are expanding exponentially with their increased popularity among users. However, this popularity has also led to an increase in reported video game addiction. There may be consumer engagement–related factors that may influence video game addiction.

**Objective:**

This study aims to empirically examine the impact of the dimensions of consumer video game engagement on video game addiction. The dimensions are dedication, absorption, conscious attention, social connection, enthusiasm, and interaction. We utilize the uses and gratifications theory to study the video game engagement dimensions as potential factors through which gamers feel gratified and engaged in video game playing. Additionally, this study incorporates the cultivation theory to investigate how video game engagement factors trigger video game addiction.

**Methods:**

A two-step process was applied for data analysis on valid cases of 176 gamers aged 15-25 years: video game addiction was specified and validated as a reflective-formative construct, and hypothesis testing was later performed using the WarpPLS on valid respondents.

**Results:**

The analysis uncovered 2 dimensions of video game engagement: social connection with *P*=.08 and interaction with *P*=.49, which did not significantly contribute to video game addiction.

**Conclusions:**

This study offers unique insights to a myriad of stakeholders, mostly psychologists and psychiatrists, who routinely prescribe behavior modification techniques to treat video game addiction.

## Introduction

### Background

Video games are one of the most popular forms of leisure activities, especially for youth (as well as other age groups) across the world [1]. The video gaming industry has come a long way since first video game, Atari, which was launched in 1972 [2]. Video game engagement has surpassed other forms of new media such as music and television owing to the rapid development of the digital gaming industry [3]. It has been estimated that digital gaming revenues rose to US $137.9 billion, with approximately 2.3 billion gamers worldwide by the end of 2018. With the rise of video gaming activities, the risk of video gaming addiction has also increased [4]. Excessive use of web-based and offline video gaming affects the gamers’ physical health and has an impact on their psychological well-being [5]. Video game addiction can cause gamers to develop a range of issues such as aggression and depression [6,7], poor academic performance [8,9], declining relations with friends and families [10,11], insomnia [12], and even self-harm [13,14].

The literature on video game engagement has identified various factors that affect video gaming addiction. Studies on demographics have identified that males, young age, singles (relationship), etc are more likely to develop video game addiction [15]. Increased monetary spending on video gaming activities is positively correlated with video game addiction alongside other factors such as the presence of heightened family resentment among gamers and an increase in the average time spent on gaming activities [16]. With respect to psychographics, the factors that predict video game addiction are depression and sociability [17], hypercompetitiveness and psychological absorption [18], and dysfunctional impulsivity [19], thereby reducing extraversion, agreeableness, emotional stability, and attractiveness [20]. Increased video game engagement and maladaptive coping has helped identify individuals at risk of transitioning into video game addiction [21].

Likewise, the phenomenon of video game engagement has been investigated in different studies across multiple application areas. For instance, Abbasi et al [22] reported how gender differences variably influence video game engagement. Gabriel et al [23] reported that rich game experiences enhance engagement in video games. Additionally, Skoric et al [24] revealed that more addicted gamers perform poorly in academic assessments. Another study found that video game addiction affects mental health, causing depression, stress, and anxiety, but video game engagement is only linked to anxiety [21]. Video game engagement has been found to have no adverse effects on gamers’ well-being [25,26], and several studies have differentiated addiction and engagement since they both result in different behavioral outcomes [27,28]. Although there are many studies on video game addiction, the research investigating the potential determinants of video game addiction through consumer video game engagement elements (eg, dedication, enthusiasm, conscious attention, absorption, social connection, interaction) remains limited.

### Study Aims and Rationale

The purpose of this study was to expand the existing research by evaluating video game addiction from the lens of video game engagement. Our primary focus in this study was to evaluate whether different dimensions of consumer video game engagement play a role in predicting video game addiction. Our study will contribute to the literature in a number of ways. First, prior studies have not accounted for engagement factors that lead to addiction [1,28,29]. Our study adds value to this overarching literature as we uncover how engagement factors influence addiction in video games. Second, this study extends the application of Uses and Gratifications Theory (UGT) as studied in media, video game, and social media [30,31] and Cultivation Theory toward developing a theoretical model that explains the role of video game engagement dimensions in predicting video game addiction. We applied UGT to video game engagement factors (conscious attention, absorption, social connection, dedication, enthusiasm, and interaction), which give rise to a cultivation effect owing to increased engagement in video game play, resulting in video game addiction. Third, prior studies considered video game addiction as a second-order construct, but its specification and validation as a reflective-formative construct remains nebulous [32]. Considering this limitation, we add to video game addiction studies literature by specifying and validating video game addiction’s construct as a reflective-formative construct.

### Theoretical Underpinning

We explored the theoretical framework informing our understanding of the different dimensions of consumer engagement and video game addiction. Different theories associated with motivation, communication, and media were used to establish the theoretical framework for this research. This research takes its theoretical underpinning from the Cultivation Theory and UGT [33,34].

#### UGT

The principle of UGT was coined in the early 1940s. This theory came into the light to answer some questions raised concerning traditional mass communication. This theory emphasizes that media is used to satisfy a user’s various needs, which motivates them to use that media [35,36]. This theory has been extensively applied to comprehend the motivation underlying media usage [37]; particularly, video games [38]. UGT is one of the most popular theories applied to assess the underlying motivation of users that engage in video gameplay [39]. With the high emphasis on video game engagement, UGT could provide a new perspective toward understanding video game addiction.

UGT consists of 3 constructs: (1) achievement, (2) enjoyment, and (3) social interaction. Achievement in the context of gaming can be defined as the yearning to achieve power and rewards as well as the desire to perform better in comparison to other gamers [40]*.* Sherry et al [39] found that challenge is the biggest motivation for playing video games as players are tempted to move through different stages quickly to complete the challenges [41]. In contrast, hardcore gamers who play video games with dedication [42] are inclined to preoccupy themselves with social comparison to flaunt their gaming achievements [43]. Enjoyment in the context refers to inherent pleasure derived from accomplishing the tasks [40], and it plays a major role when it comes to assessing an individual’s desire to use a specific information system [44]. Social interaction in web-based gaming opens new channels of communication since gamers have an opportunity to socialize and cultivate existing or new relationships. Web-based games, therefore, provide an avenue to gamers, especially those who experience loneliness and low self-esteem, thus supporting individual desire to play video games [45]. Similarly, the UGT perspective accentuates the self-indulgent needs and interests that influence the motivation to engage in digital games [46]. Thus, UGT appears to be a relevant theory for this research since video game players actively indulge in video games, thereby leading to video game addiction.

#### Cultivation Theory

The explanation of mass media as cultivation was introduced by Gerbner [47]. The Cultivation Theory uncovers how mass media shapes the perceptions of people [48]. The Cultivation Theory suggests that exposure to media influences viewers’ interpretation of reality [49]. Research has already demonstrated this phenomenon by investigating individuals’ social settings and observing changes in people’s attitudes and social norms [50,51]. Therefore, the media carries the capacity to enculturate the masses to adapt to the changing environment around them [52].

The Cultivation Theory provides a wide lens through which long-term effects of media can be examined. The Cultivation Theory has mostly been applied to traditional forms of media such as television. Aligned with the Cultivation Theory assumptions with respect to television (similarly, if this concept is applied to video gaming), this theory posits that through continuous exposure to the video gaming world, player’s views of his real-world will become more similar to that of his gaming world [53]. For that purpose, Van Mierlo and Van den Bulck [54] argued that video games have become so authentic that they have started to mirror reality, thereby making cultivation possible [34]. Hence, video game players engage with different forms of synthetic media such as in-game challenges, stories, and characters, and this involvement in the video games is associated with concentrated attention [23], because of which the game player identifies himself with the role-played character and creates a visually epitomized virtual identity [55]. Thus, video games have started resembling reality, enabling the cultivation of an immersive reality [56]. Seah and Cairns [57] have also reported that the more immersed a gamer becomes, the immersion may lead to video game addiction.

### Consumer Video Game Addiction

The massive popularity of video games has significantly changed the gaming industry as it is one of the most booming entertainment businesses [58]. Several studies have documented the risks and effects of video game addiction. A recent meta-analytic review, including 136 articles with 130,000 participants from both Eastern and Western cultures, found that video game addiction is likely to cause anxiety, depression, social phobia, and scholastic decline [59]. The study suggests that harmonious passion and obsessive passion predict different ways to engage in massively multiplayer online role-playing game (MMORPG). The study further confirms that passion is a valuable paradigm to recognize diverse motivational patterns expressed by MMORPG players [60]. Wittek et al [61] posit that video game addiction has a negative relationship with conscientiousness and a positive association with neuroticism. Hull et al [17] report that the social features of video gaming, distortion of time perceptions, and happiness levels significantly predict video game addiction. Apart from this, video game addiction is also reported to be associated with lower psychological functioning, unsatisfactory academic performance, increased alcohol consumption, sleep deprivation, aggression and depression, social disconnectedness, and increased caffeine consumption [17,62-64].

### Consumer Video Game Engagement

Consumer engagement is the interaction between a customer and an organizational entity through different channels whereas engagement in consumer game-playing is a concept that signals task-oriented consumer behavior in the game setting [65]. Video games are purposely intended to draw the attention of its consumer base to ensure that video game consumers will evolve into frequent game players [66,67]. This appeal is primarily engendered by the engaging experiences video games are intended to deliver [68]. These experiences are coined as playful video game consumption experience and have led to behavioral changes among its consumer base [69].

Previous studies deem engagement as a multidimensional construct that comprises of subconstructs such as immersion [70,71], presence [72], flow [73], fun [74], enjoyment [72,75], fun [74], and absorption [76]. Hollebeek et al [77] also suggest that the construct *engagement* is a multidimensional concept comprising of 3 dimensions: (1) cognitive, (2) emotional, and (3) behavioral. Researchers also accentuate that different dimensions of consumer engagement are interrelated [78]. For instance, Brodie et al [79] identified that emotional involvement can increase other video game engagement dimensions (ie, cognitive and behavioral).

Bouvier et al [41] identified 4 types of gaming engagement behaviors, that is, (1) environment-directed behavior, (2) social-directed behavior, (3) self-directed behavior, and (4) action-directed behavior. They further explain that environment-directed behavior refers to the participation of players oriented toward the setting or the ecological backdrop in the game. In contrast, social participation alludes to the social links present in the game. Games offer players the opportunity to develop and foster their social relationships with other players, thereby establishing social links in the virtual environment. Self-directed behavior refers to the relationship between players and their virtual character. For instance, we often see players have a preference to personalize their virtual presence such as customizing their avatar or opting for accessories for reasons other than performance. Lastly, action-directed behavior refers to goal-directed measures taken by players in different in-game situations. Players act for many reasons such as seeking to quickly move between stages, gain different experiences, and accomplish challenges.

Abbasi et al [22] report multidimensionality within video game engagement and demonstrates that the construct comprises of 6 dimensions, that is, (1) dedication, (2) absorption, (3) conscious attention, (4) social connection, (5) enthusiasm, and (6) interaction. This study aims to determine which of these dimensions can result in addiction to video games, which may provide empirical evidence in this sector.

### Game Addiction and Game Engagement: Are They Similar or Distinct?

Video game addiction and video game engagement were previously used interchangeably [24]. However, recently, these 2 terms have been considered as 2 separate entities [15]. Distinguishing gaming addiction from gaming engagement has been a challenge for researchers [15]. Several studies have approached this concern by differentiating game addiction from game engagement; first, Charlton and Danforth [80] showed that a gaming addict group spends twice the hours as a highly engaged gamers group in playing games each week. In another study, game addiction scores were negatively related to emotional stability; however, gaming engagement scores remained unchanged [20]. Charlton and Danforth [80] also reported a considerable difference between video game addiction and video game engagement. They posited that addiction should not be confused with active engagement as addiction includes withdrawal symptoms, that is, anxiety and guilt. Brunborg et al [15] also distinguished video game addiction from video game engagement through a self-administered questionnaire. In their study, engaged gamers showed salience and mood modification, whereas addicted gamers exhibited anxiety, apprehension, touchiness, and isolation. The key differentiation between addiction and engagement included the range of adverse consequences experienced by the consumers [81,82]. Video game addiction is possibly linked to a range of adverse effects such as mental, physical, and social deterioration, but this is not the case in high engagement [82]. Video game addiction is mainly linked with growing signs of stress, anxiety, and depression [83].

### Dimensions of Consumer Video Game Engagement

Dedication is the affective commitment of players toward video games [84]. Kallio et al [85] reported that some gamers play for pleasure, whereas others play to form a connection with the game. Players who are committed and loyal spend more time playing social games, ensuring that they are dedicated to the video game [85]. Similarly, it has been found that hardcore gamers on average play more than an hour per day [86,87], spending most of their leisure time dedicated to gaming activities [42,88]. On the contrary, casual gamers spend less time playing games since they play in small bursts and have a casual attitude toward gaming [89]. Conrad [90] found that games tended to be addictive if played for more extended periods. Griffiths [91] assessed the consequences of excessive gaming and found that players experienced behavioral addiction symptoms, including salience, mood modification, and tolerance. Van Rooij et al [92] reported that teenagers who spend an average of 55 hours per week on gaming tended to develop depressive moods, loneliness, social anxiety, and negative self-esteem. On the basis of the abovementioned relationships, we posit the following hypothesis:

H1: Dedication positively predicts video game addiction in a video game player.

Absorption is defined as the degree of involvement and immersion in a given activity [93]. Csikszentmihalyi [94] reports that absorption in an activity is regarded as “flow experience,” and video games encourage a state of flow and learning in gamers [95]. Players in this state immerse themselves and become absorbed in their activity. This way, they become oblivious to their surroundings, which narrows their focus of situation awareness. De Pasquale et al [96] reported that excessive absorption of video games leads to neglecting the surrounding environments. Furthermore, when trying to curb video game playing, absorption has been correlated with anxiety, irritability, and emotional fragility. Excessive video game absorption is unhealthy and can potentially lead to addiction [57,97]. Based on these relationships, we posit the following hypothesis:

H2: Absorption in video games significantly predicts video game addiction in a video game player.

Conscious attention is the attention and time allocated toward a given activity [98-100]. Consciousness is a “complex system that has evolved in humans for selecting information from this profusion, processing it, and storing it” and determines “what to pay attention, how intense, and for how long” [101]. In the state of flow, an individual experiences conscious processes followed by attentional processes, which require concentration and unilateral focus [101]. The individual operates at full capacity [102], devotes full attention to achieve goals [94], and goes through the distortion of temporal experiences [101]. Haladjian and Montemayor [103] report that conscious attention lets us intermingle with our complicated surroundings and enables us to remain involved in the task at hand. Similarly, consistent, conscious attention over a considerable period makes the player more absorbed. Therefore, conscious attention and video game consumption carry addictive tendencies and lead to addiction [57,97]. We further posit the following hypothesis:

H3: Conscious attention predicts video game addiction in a video game player.

Social connection is the relationship people have with other individuals or groups [104]. Web-based gamers may shape meaningful relationships through social connections made during interactive gameplay [105]. Digital games can often create unrestrained social environments where players connect and form connections [106,107]. According to the augmentation hypothesis, people with an active social life also endeavor to build social ties in the virtual world [108]. Sioni et al [109] reported that video game addiction is very likely to occur owing to social phobia. Cole and Griffiths [110] suggested that people often try to develop long-term friendships based on their gaming experiences, and this socialization aspect tends to keep them motivated to continue playing. On the contrary, Peters and Malesky Jr [111] reported that gamers looking for social connections in gaming contexts may struggle to form tangible social connections outside gaming environments. It has additionally been found that people who cannot develop real-life connections tend to develop web-based virtual connections, and people who have social anxiety as well as depression also tend to use web-based media, which has been associated with addictive symptoms [15]. Accordingly, we put forward the following hypothesis:

H4: Social connections significantly predict video game addiction in a video game player.

Enthusiasm is defined as the extent of eagerness and interest toward a given activity [104,112]. Enthusiasm is the inner driver that motivates individuals to play video games. Enthusiasm encourages individuals to overcome obstacles to reach higher levels in games [113]. Griffiths [91] adds that one can differentiate between enthusiasm and addiction since enthusiasm contributes to life, whereas addiction takes away life. Although enthusiasm and addiction can most certainly be different phenomena and experiences, it can be fair to conclude that excessive enthusiasm for a particular video game can potentially lead to addiction. Hence, we put forward the following hypothesis:

H5: Enthusiasm significantly predicts video game addiction in a video game player.

**Figure 1:**
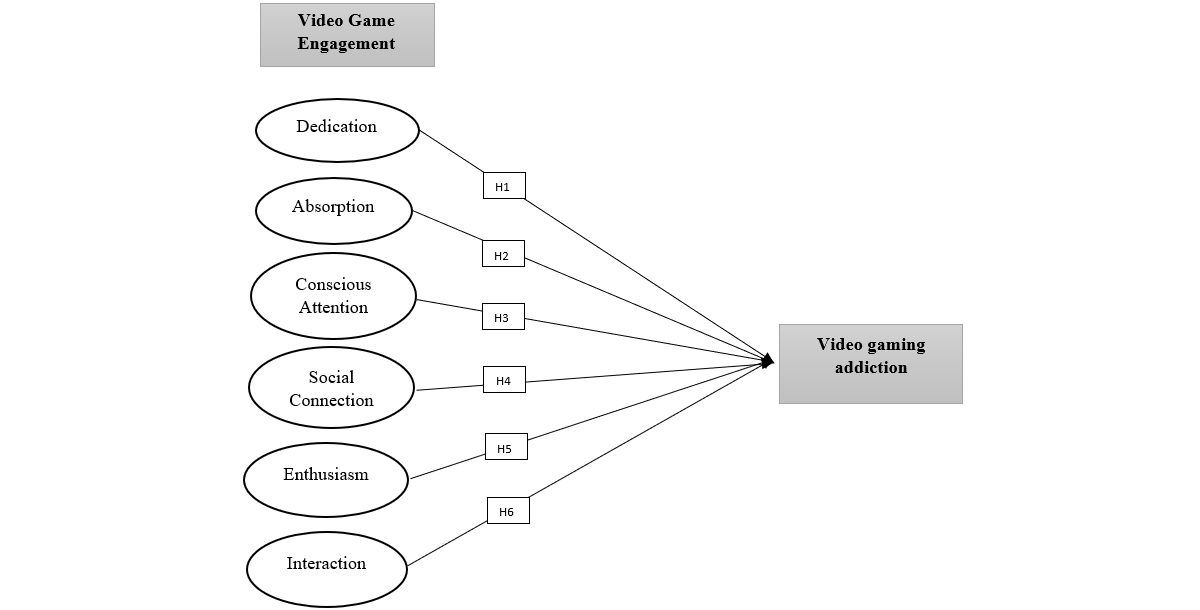
Study conceptual model.

Interactions reflect the degree of involvement that individuals and other entities have in virtual game settings. So et al [112] report that interactions signify how players interact with synthetic game components. Interaction is positively correlated with consumer video game engagement [114] since interactive experiences influence the degree of consumer engagement [115,116]. Weibel et al [107] reported that gamers actively seek interaction with other players; this social element leads to increased duration of gameplay and heightened engagement levels. This has the potency to result in video game engagement [117]. Griffiths [91] reports that excessive interaction between human-machine components can influence different aspects of addiction, including mood, tolerance, withdrawal symptoms, etc. Similarly, it has been found that social interaction is one of the triggers of addiction [118]. Thus, we put forward the following hypothesis (see Figure 1 for the full study model and hypotheses):

H6: Interaction positively predicts video game addiction in a video game player.

## Methods

### Study Design

This study was quantitative and cross-sectional. This approach is relatively fast in obtaining responses to develop or validate new theories [119]. Following this approach, we required a survey to collect the data (Multimedia Appendix 1). Hence, we designed the survey into 2 units: the first unit of the questionnaire included the participants’ demographic profiles regarding their video gaming consumption patterns. Questions in the second unit were related to the constructs of the theoretical model, including engagement and addiction. As previously mentioned, video gaming engagement comprised 6 dimensions, whereas video gaming addiction was formulated as 7 dimensions of the second-order construct. The items measuring the video game engagement dimensions were adapted [22,28,120]. The items assessing the construct of video game addiction were adapted [121]. The questionnaire items were evaluated on a 5-point Likert Scale from 1 (strongly disagree) to 5 (strongly agree). Hair Jr et al [122] recommended applying the G*power analysis tool when the sample frame is unknown. Using the power analysis, we set the input parameters as follows: effect size (f²)=0.15, α error probability=.05, power (1-β error probability)=.95, and predictors=6, which calculated the total sample size of 146 for the study model to conduct partial least squares-structural equation modeling (PLS-SEM) analyses. To sample the participants, we considered purposive sampling owing to its popularity in social sciences and the ability to obtain a sample that can represent the population [123].

This study was carried out among gamers who play in gaming zones (a gaming zone is a venue or an entertainment service provider, which facilitates users to play competitive games in groups to compete with each other) [124] or in organized tournaments at institutions located in the twin cities of Islamabad and Rawalpindi. The only inclusion criterion was if they play video games (eg, Counter Strike–Global Offensive, PlayerUnknown’s Battlegrounds, Call of Duty, Fortnite) daily for a minimum of 1 hour. The main reason for selecting 1 hour as the minimum criterion for sample selection was that playing video games in gaming zone/cafe is an expensive activity in low-income countries such as Pakistan because gamers are charged about PKR 300-500 (US $1.5-$3) per hour/session, whereas the per capita income in Pakistan is only US $1516. Second, parents are authoritarian in Pakistan and they may limit their children from longer screen time as the age bracket from 15 to 20 years is considered as the prime age for education and career development [125]. Third, gamers older than 21 years could be engaged in some jobs with their studies to meet their needs and wants.

While collecting the data, we carefully recruited gamers who played Counter Strike–Global Offensive, PlayerUnknown’s Battlegrounds, Call of Duty, and Fortnite and proceeded for data collection. Using the self-administered approach, we distributed 250 questionnaires and 190 were returned. We assessed the data quality and removed incomplete and biased responses (ie, using the particular response pattern) (Multimedia Appendix 2). Finally, we had 176 valid responses to continue with data analyses; see Table 1 for participants’ details.

The structural equation modeling approach was applied for analysis, which is a well-rounded multivariate statistical analysis approach [126,127]. Structural equation modeling appears to be the most suitable approach because the research variables consist of both formative and reflective constructs [128]. For PLS-SEM analysis, WarpPLS software by Kock [129] was employed in this research.

**Table 1:** Characteristics of the participants (N=176).

Characteristics		Values, n (%)
**Gender**		
	Male	128 (72.7)
	Female	48 (27.3)
**Age (years)**		
	15-20	53 (30.1)
	21-25	123 (69.9)
**Education**		
	Higher secondary education	45 (25.6)
	Bachelor’s	65 (36.9)
	Master’s	66 (37.5)
**Expenditures on video games daily (exchange rate: 1 USD=PKR 153.314 in December 2019)**		
	PKR 0-500	88 (50)
	PKR 500-1000	58 (33)
	PKR 1000-5000	20 (11.4)
	Above PKR 5000	10 (5.7)
**Device used for gaming**		
	Play station	59 (33.5)
	Cybercafé	45 (25.6)
	Xbox	26 (14.8)
	Others	46 (26.1)
**Games played the most**		
	Counter Strike–Global Offensive	55 (31.3)
	PlayerUnknown’s Battlegrounds	29 (16.5)
	Call of duty	68 (38.6)
	Fortnite	24 (13.6)
**Time spent on video games daily (hours)**		
	0-1	72 (40.9)
	1-2	65 (36.9)
	2-4	39 (22.2)

### Assessing the Reflective Constructs

In the measurement model’s assessment, first, the reflective constructs were assessed in which the construct’s outer loadings, Cronbach alpha, composite reliability, and convergent validity were evaluated. According to the criteria, the outer loadings should be greater than at least 0.40, the Cronbach alpha value should be greater than .70, the composite reliability should be greater than 0.70, and the convergent validity should be greater than 0.5 [126,128]. The values for all the measures met the benchmark, thereby ensuring the reliability and the validity of the constructs; hence, the measurement model of the reflective measures was satisfactorily fulfilled (Table 2).

Additionally, the discriminant validity was evaluated applying the Fornell and Larcker (1981) criterion. The criterion suggests that the square root of the average variance extracted of each dimension must be larger than its corresponding correlation coefficient. Heterotrait-monotrait ratio of correlations is another criterion that is the most recent and recommended validity test to assess the discriminant validity for reflective constructs [130]. The heterotrait-monotrait ratio of correlations values are deemed to be satisfactory if lower than 0.90 and best if lower than 0.85. The results in Table 3 and Table 4 reported that discriminant validity had attained satisfactory values.

**Table 2:** Measurement model: first-order constructs.

Scale, items			Loading		Composite reliability			Cronbach alpha			Average variance extracted
**Conscious attention**						0.935			.916		0.707
	Item 1	0.84									
	Item 2	0.872									
	Item 3	0.855									
	Item 4	0.902									
	Item 5	0.842									
	Item 6	0.724									
**Absorption**						0.93			.906		0.728
	Item 1	0.838									
	Item 2	0.866									
	Item 3	0.895									
	Item 4	0.863									
	Item 5	0.802									
**Dedication**						0.928			.903		0.72
	Item 1	0.851									
	Item 2	0.863									
	Item 3	0.856									
	Item 4	0.842									
	Item 5	0.83									
**Enthusiasm**						0.893			.85		0.628
	Item 1	0.688									
	Item 2	0.832									
	Item 3	0.844									
	Item 4	0.816									
	Item 5	0.771									
**Social connection**						0.9			.833		0.75
	Item 1	0.865									
	Item 2	0.871									
	Item 3	0.862									
**Interaction**						0.93			.906		0.727
	Item 1	0.832									
	Item 2	0.878									
	Item 3	0.866									
	Item 4	0.858									
	Item 5	0.827									
**Salience**						0.91			.851		0.771
	Item 1	0.873									
	Item 2	0.872									
	Item 3	0.889									
**Tolerance**						0.893			.82		0.736
	Item 1	0.841									
	Item 2	0.894									
	Item 3	0.837									
**Mood modification**						0.902			.836		0.753
	Item 1	0.859									
	Item 2	0.862									
	Item 3	0.883									
**Relapse**						0.913			.857		0.778
	Item 1	0.879									
	Item 2	0.897									
	Item 3	0.87									
**Withdrawal**						0.897			.828		0.745
	Item 1	0.831									
	Item 2	0.908									
	Item 3	0.848									
**Conflict**						0.912			.855		0.775
	Item 1	0.873									
	Item 2	0.896									
	Item 3	0.872									
**Problem**						0.952			.924		0.869
	Item 1	0.928									
	Item 2	0.958									
	Item 3	0.909									

**Table 3:** Discriminant validity using Fornell and Larcker criterion.

Dimension	Conscious attention	Absorption	Dedication	Enthusiasm	Social connection	Interaction	Salience	Tolerance	Mood modification	Relapse	Withdrawal	Conflict	Problem
Conscious attention	0.841												
Absorption	0.500	0.853											
Dedication	0.402	0.538	0.849										
Enthusiasm	0.439	0.496	0.544	0.792									
Social connection	0.313	0.440	0.275	0.601	0.866								
Interaction	0.487	0.454	0.405	0.573	0.663	0.853							
Salience	0.402	0.510	0.462	0.566	0.527	0.565	0.878						
Tolerance	0.336	0.456	0.373	0.479	0.435	0.435	0.614	0.858					
Mood modification	0.323	0.310	0.239	0.403	0.300	0.322	0.395	0.461	0.868				
Relapse	0.248	0.379	0.288	0.401	0.288	0.265	0.499	0.413	0.587	0.882			
Withdrawal	0.276	0.334	0.334	0.304	0.221	0.229	0.397	0.424	0.535	0.736	0.863		
Conflict	0.365	0.201	0.248	0.212	0.146	0.133	0.241	0.291	0.447	0.392	0.535	0.88	
Problem	0.265	0.357	0.353	0.296	0.195	0.217	0.333	0.415	0.286	0.366	0.374	0.426	0.932

**Table 4:** Discriminant validity using heterotrait-monotrait ratio of correlations.^a^

	Conscious attention	Absorption	Dedication	Enthusiasm	Social connection	Interaction	Salience	Tolerance	Mood modification	Relapse	Withdrawal	Conflict	Problem
Conscious attention													
Absorption	0.550												
Dedication	0.441	0.596											
Enthusiasm	0.499	0.571	0.625										
Social connection	0.357	0.507	0.316	0.715									
Interaction	0.537	0.504	0.448	0.649	0.763								
Salience	0.455	0.580	0.526	0.668	0.625	0.645							
Tolerance	0.387	0.529	0.433	0.576	0.525	0.504	0.733						
Mood modification	0.371	0.355	0.275	0.480	0.359	0.370	0.468	0.558					
Relapse	0.278	0.430	0.327	0.470	0.341	0.301	0.584	0.492	0.692				
Withdrawal	0.314	0.387	0.385	0.363	0.267	0.268	0.475	0.516	0.647	0.876			
Conflict	0.410	0.227	0.282	0.254	0.173	0.152	0.281	0.347	0.529	0.458	0.634		
Problem	0.290	0.390	0.386	0.339	0.221	0.237	0.374	0.476	0.325	0.411	0.427	0.480	

^a^Heterotrait-monotrait ratio of correlations: good if <0.90; best if <0.85.

### Assessing the Formative Constructs

**Figure 2:**
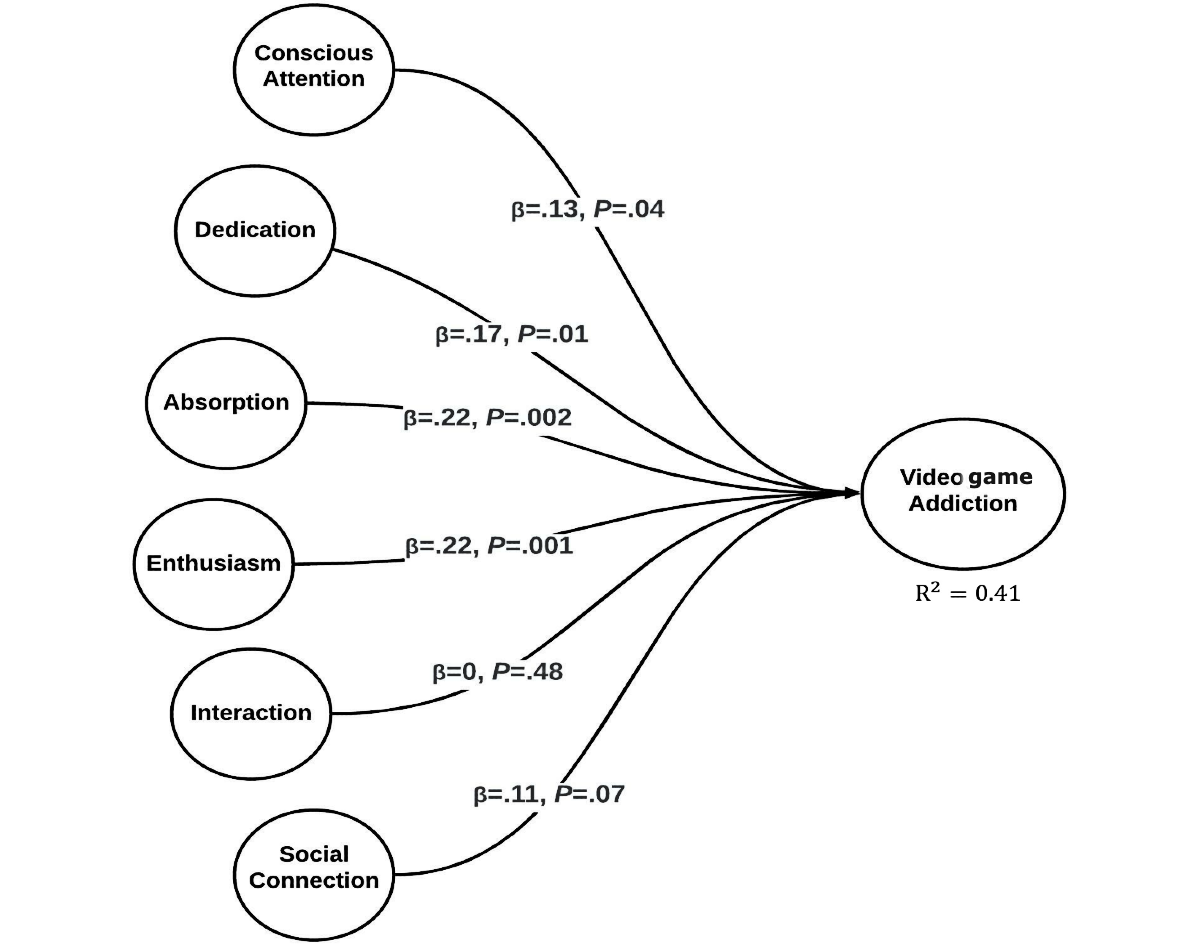
The empirical-based study model.

To create the second-order formative construct, that is, video game addiction, we employed the two-stage approach of Becker et al [131] to calculate the latent variable scores of first-order reflective constructs of salience, problem, withdrawal, mood modification, relapse, conflict, and tolerance. Afterwards, the latent variables scores were used to create the video game addiction construct as specified in Figure 1 and Figure 2. The quality checks for assessing the formative construct were quite different from those for assessing reflective constructs. Assessing the formative construct for reliability and validity, researchers suggest to use variance inflation factor (the values should be <5 or <3.3, the most restrictive one) and calculate the weights and its significance [29,132]. We analyzed the formative construct and reported the results in Table 5, thereby showing the formative construct’s validity. It met the threshold values, that is, variance inflation factor values are lower than 3.3 and indicator weights are significant.

**Table 5:** Evaluation of the formative measurement model on the second-order constructs of video game addiction.

Items	Scale type	Weights	Significance	Full collinearity
Salience	Formative	0.188	.005	2.383
Tolerance	Formative	0.195	.004	1.987
Mood modification	Formative	0.205	.003	1.892
Relapse	Formative	0.223	.001	2.867
Withdrawal	Formative	0.222	.001	2.744
Conflict	Formative	0.178	.008	1.780
Problems	Formative	0.167	.01	1.485

## Results

PLS-SEM analysis was conducted in 2 stages comprising the measurement model and structural model assessment. Once the reliability and validity of the reflective and formative constructs were ensured, researchers recommended evaluating the structural model through path coefficients, effect size, *t* value and calculated the predictive relevance, that is, Q^2^ and R^2^ coefficient value for the endogenous constructs [28,29,128]. WarpPLS was used to validate the hypotheses. For H1, the analysis results revealed a significant relation with B=0.129, *P*=.04, and *t* statistics of 1.76 with *f^2^*=0.056. Hence, H1 was supported. For H2, the results revealed a significant impact of absorption on the consumer video game addiction with B=0.215, *P*=.002, and *t* statistics of 2.98 with *f^2^*>=0.113. For H3, the results were supported with B=0.166, *P*=.01, and *t* statistics of 2.27 with *f^2^*=0.079. For H4, the hypothesis was also supported with B=0.220, *P*=.001, and *t* statistics of 3.05 with *f^2^*=0.118. For H5, the analysis results revealed that the relationship between both the constructs was insignificant with B=0.107, *P*=.08, and *t* value of 1.44. Hence H5 was rejected. Similarly, interaction with consumer video game addiction was also insignificant with B=–0.003, *P*=.49, and *t* value of 0.04. We also calculated the R^2^ and Q^2^ for the endogenous construct, that is, video game addiction, which resulted in R^2^=0.409 and Q^2^=0.413. Our results showed that the study model has sufficient explanatory capacity and predictive relevance. Figure 2 and Table 6 show the results of the structural model assessment.

**Table 6:** Structural model assessment.

Dimensions of video game addiction	B	SE	*t* value^a^	*P* value	*F^2^*
Conscious attention	0.129	0.073	1.76	.04	0.056
Absorption	0.215	0.072	2.98	.002	0.113
Dedication	0.166	0.073	2.27	.01	0.079
Enthusiasm	0.220	0.072	3.05	.001	0.118
Social connection	0.107	0.074	1.44	.08	0.045
Interaction	–0.003	0.075	0.04	.49	0.001

^a^Degree of freedom not provided because WarpPLS software was used.

## Discussion

### Principal Findings

In this study, we intended to empirically test the theoretical model illustrating the different dimensions of video game engagement, that is, (1) dedication, (2) absorption, (3) conscious attention, (4) social connection, (5) enthusiasm, and (6) interaction in a digital game, that predict consumer video game addiction. The underlying constructs were supported by theoretical underpinnings from the Cultivation Theory [47] and UGT [37]. Our results indicated that 4 out of 6 dimensions (ie, dedication, absorption, conscious attention, and enthusiasm in consumer video game engagement) significantly predict consumer video game addiction; however, social connection and interaction in consumer video game engagement had no significant effect on video game addiction.

Previous research suggests various personal, emotional, and psychological needs that contribute to overindulgence in video gameplay [133,134]. It has also been reported that gaming environments provide safe and interactive avenues for individuals who experience social disconnection or isolation in real-life environments [15]. This is also a means of open interaction for such individuals since the consequences for actions in gaming environments are minimal; therefore, the fear of interaction subsides [107]. Peer pressure in social groups is also a significant reason for excessive indulgence in video games. The media hype of video games have led some people to assume that behavioral problems are triggered by video games and contribute to video game addiction [135]. Further, rigorous advertising on different media plays a role in convincing individuals to play video games and experience instant gratification and other psychological needs. Cultivation Theory unravels the grounds explaining why individuals are addicted to video gaming, primarily because increased exposure to video games carries negative consequences. Such results are consistent with those reported in previous literature, where addiction contributes to different problems in the individual’s personality and social life [136].

H1 showed the relationship between dedication and the resulting video game addiction. The results supported that there is a positive relationship between the 2 constructs. They showed that dedicated players (and attached) to the gameplay for long hours put in more energy, commitment, and loyalty than casual players [85,137]. H2 showed the relationship between absorption and video game addiction. Our results supported the positive relationship between the 2 constructs—consistent with that reported in previous literature [138]. They indicated that absorption leads to deep involvement in the game, which results in neglecting the surrounding environments and resulting in various problematic behaviors [96]. H3 showed the relationship between conscious attention and video gaming addiction. The results supported this testable statement (showed a positive effect between conscious attention and video gaming addiction). Through conscious attention, players tend to develop deep concentration [101], which results in neglecting the surroundings [103], leading toward video game addiction. H4 showed the relation between enthusiasm and video gaming addiction. As reported previously by Seay and Kraut [139], we found that excessive consumption of entertaining electronic gadgets can create interference in the real lives of the individuals.

Social connection and interaction were hypothesized to positively affect video game addiction (H4 and H6, respectively). However, both the constructs had no significant relationship with video game addiction. These results seem to contradict the prior literature [109,110,140-142]. Based on the context of Pakistan, this study is not in line with these 2 concepts. Pakistan, being a collectivist society, encourages people to interact socially, and excessive video gaming tends to make them socially isolated. The social connections developed during video gaming sessions seem to have little or no effect on video gaming addiction. The sense of isolation due to excessive use of technology has also been confirmed in different studies concerning internet usage [143,144]. This contradictory finding in our study opens a new door for future research as to why video gaming social connectedness does not predict video gaming addiction in Pakistan and whether the findings of this study can be applied to other contexts. We also concluded that the interaction does not predict video game addiction. Sedig and Parsons [114] report that video games are purposefully designed for a highly interactive experience. Although interactive video games lead to a more profound interest and increased engagement, they do not necessarily result in gaming addiction. Additional research is required to investigate this finding further.

### Theoretical Implications

The results of this study contribute to the understanding of the Cultivation Theory in the context of video games, that is, video game addiction [145]. While the focus of Cultivation Theory has mainly been restricted to the effects of video-based media, for example, television and films, this research applies it in the context of video games, especially how video game engagement factors cultivate addiction in video game players. This expands the scope of the theory to media that is not merely passive but also interactive in video games. This may help future researchers understand how behaviors, especially negative ones, are influenced by video game material, that is, video game engagement dimensions. Similarly, this study also helps expand the scope of the UGT. It does this by exploring the different video game engagement factors comprising absorption, conscious-attention, dedication, enjoyment, and social interaction in video games as user’s gratifications are derived from video game play. This study’s conceptual model highlights factors that motivate users of pleasure-oriented information systems, specifically video games, to continue prolonged usage. Specifically, this study offers dedication, absorption, conscious attention, and enthusiasm as significant, positively impacting factors that lead to addiction. This adds another dimension to the understanding of video game addiction by combining cultivation theory, UGT, and these unique factors that lead to habit-building that turns into an addiction. Moreover, this study offers insight into the reasons underlying video game usage, mostly hedonic, and how it is reinforced due to a specific gameplay characteristic. In doing so, it helps highlight the reasons underlying engagement with pleasure-oriented information systems to construct a holistic conceptual model where usage is observed as a form of meeting certain needs (UGT) and shaping worldviews (cultivation theory). Previously, authors conceptualized and specified the gaming addiction construct as a reflective-formative construct, but its empirical validation remains nebulous to date [32]. Addressing this knowledge gap, we employ the hierarchical component model approach as recommended by Sarstedt et al [132] to operationalize, specify, and validate the construct of video game addiction as a reflective-formative construct.

### Practical Implications

This study offers valuable insight for practitioners in game development, psychology, and social policy as well. First, an understanding of why consumers continue to use games up to the point of addiction can highlight factors that help make games engaging. This can be used to cultivate brand loyalty as game developers roll out updates of their games. Moreover, the insignificant correlation between social connections and video game addiction will help developers understand how social bonds may not facilitate engagement for specific regions such as Pakistan, in this case. At the same time, this information can also be used to curb factors that turn repeated or prolonged usage into a harmful addiction. This can help game developers ensure that they are producing games conducive to healthy usage to meet health care and social concerns. Similarly, these findings can be used by mental health practitioners who aim to find treatment solutions for video game addictions. By understanding the aspects of video game play that leads to addiction, these practitioners may be better able to prescribe changes to playing behavior and patterns to reduce addiction. In terms of policymaking, this study helps policymakers understand what aspects of video game playing lead to the development of problematic real-life behaviors that are considered unhealthy or are discouraged.

### Conclusion

This study seeks to examine the individual effect of different dimensions of video gaming engagement on video game addiction. The findings of our study add value to the existing literature. The factors that lead to video gaming addiction can be empirically stated through the findings of this study. This research also provides valuable insights into the consumption pattern of video gamers. Future research directions could involve incorporating the psychographic mechanisms as moderators such as personality traits, values and attitudes, interests, lifestyles, and gender in understanding video game addiction [146]. Additionally, the theoretical model could be applied to investigate the antecedents of video gaming engagement, that is, the factors that cause people to engage in video gaming consumption and that video game interaction does not have a significant relationship with video gaming addiction. However, the literature suggests that video games are designed to have an interactive interface to engage consumers. In this regard, a mediating mechanism of absorption, immersion, and pleasure could be empirically tested to show how the interactive interfaces convert the video gaming engagement into video game addiction.

## Appendix

Multimedia Appendix 1 Questionnaire used in this study.

Multimedia Appendix 2 Data analysis file.

## Abbreviations

MMORPG: massively multiplayer online role-playing game
PLS-SEM: partial least squares-structural equation modeling
UGT: uses and gratifications theory
