# Supplementary material for: Predicting Video Game Addiction Through the Dimensions of Consumer Video Game Engagement: Quantitative and Cross-sectional Study
Source: JMIR Serious Games. 2021 Nov 26;9(4):e30310. doi: 10.2196/30310 (PMC8665386; doi:10.2196/30310)
Supplement: Multimedia Appendix 1 [file games_v9i4e30310_app1.docx]

| Construct  **Multimedia Appendix 1. Questionnaire used in this study.** | Dimension | Items |
| --- | --- | --- |
| Consumer Video game engagement | | |
| Cognitive engagement (CE) | Conscious attention (CA) | I like to learn more about the video-game.  I notice information related to the video-game.  I pay a lot of attention to anything about the video-game.  I keep up with things related to the video-game.  Anything related to this video-game grabs my attention.  I concentrate on the video-game’s story for a long time Absorption |
|  | Absorption (AB) | When I am playing a video-game, I forget everything else around me.  Time flies when I am playing the video-game.  When I am playing a video-game, I get carried away.  When I am playing this video-game, I feel immersed.  I feel happy, when I am playing this video-game intensely. |
| Affective engagement (AE) | Dedication (DE) | This video-game inspires me.  I am enthusiastic about playing this video-game.  I am proud of playing this video-game.  I find this video-game full of meaning and purpose.  I am excited when playing this video-game. |
|  | Enthusiasm (EN) | I spend a lot of my discretionary time playing this video-game.  I am heavily into playing this video-game.  I am passionate about playing this video-game.  I enjoy spending time playing this video-game.  I try to fit playing this video-game into my schedule. |
| Behavioral engagement (BE) | Interaction (IN) | In general, I like to get involved in the discussions about this video-game playing.  I am someone who enjoys playing this video-game with others like-minded video-game players.  I am someone who likes actively participating in the discussions about this video-game playing.  In general, I thoroughly enjoy exchanging ideas on this video-game with other video-game players.  I often participate in activities relating to this video-game. |
|  | Social connection (SC) | I love playing this video-game with my friends.  I enjoy playing this video-game more when I am with others.  Playing this video-game is more fun when other people around me play it too. |
| Video game addiction | | |
|  | Salience | Did you think about playing a game all day long?  Did you spend much free time on games?  Have you felt addicted to a game? |
|  | Tolerance | Did you play longer than intended?  Did you spend increasing amounts of time on games?  Were you unable to stop once you started playing? |
|  | Mood Modification | Did you play games to forget about real life?  Have you played games to release stress?  Have you played games to feel better? |
|  | Relapse | Were you unable to reduce your game time?  Have others unsuccessfully tried to reduce your game use?  Have you failed when trying to reduce game time? |
|  | Withdrawal | Have you felt bad when you were unable to play?  Have you become angry when unable to play?  Have you become stressed when unable to play? |
|  | Conflict | Did you have fights with others (e.g., family, friends) over your time spent on games?  Have you neglected others (e.g., family, friends) because you were playing games?  Have you lied about time spent on games? |
|  | Problems | Has your time on games caused sleep deprivation?  Have you neglected other important activities (e.g., school, work, sports) to play games?*  Did you feel bad after playing for a long time? |
